# Supplementary material for: Navigating the Landscape: A Comprehensive Review of Current Virus Databases
Source: Viruses. 2023 Aug 29;15(9):1834. doi: 10.3390/v15091834 (PMC10537806; doi:10.3390/v15091834)
Supplement: Supplementary file 1 [file viruses-15-01834-s001.zip › viruses-2515840-supplementary.pdf]

Supplementary Materials:

Table S1. This table presents the status of virus databases and tools reviewed by Sharma et al. in 2015 [22]. For each database, data on availability, last update, URL, and citation are listed. Of the 50 virus databases listed in the original paper, 20 are not reachable, and only 11 have been updated since 2022. The number of citations were collected in January 2023. [a]: all these databases were merged into BVBRC; [b]: the website does not contain the promised data; [c]: the website does not load anymore (tested for 5 minutes, on different days); [d]: no update since creation; "–" = Not Available

Website	Acc	Up-date	URL	Num. of citation
AVPdb	Yes	2013	http://crdd.osdd.net/servers/avpdb	171 [91]
bNAber	No	-	http://bnaber.org	88 [92]
CAPIHd	No	-	http://bioinfo-dbb.nhri.org.tw/capih	71 [93]
CoVDB	No[c]	-	http://covdb.microbiology.hku.hk	62 [94]
DPVweb	Yes	2013	http://www.dpvweb.net/	92 [95]
EpiFlu	No	-	http://platform.gisaid.org	3890[48–50]
euHCVdb	Yes	2011	http://euhcvdb.ibcp.fr	161 [96]
EuResist	Yes	2023	http://www.euresist.org	400 [55]
FLAVIdB	No[c]	-	http://cvc.dfci.harvard.edu/flavi/	24 [97]
Flavitrack	No[c]	-	http://carnot.utmb.edu/flavitrack	26 [98]
HBVdb	Yes	2023	https://hbvdb.lyon.inserm.fr/HBVdb/	177 [57]
HBVRegDB	No[c]	-	http://lancelot.otago.ac.nz	40 [99]
HERVd	Yes	2021	http://herv.img.cas.cz	110 [100]
HESAS	Yes	2004	http://www.primate.or.kr/HESAS	21 [101]
HIPdb	Yes	2013[d]	http://crdd.osdd.net/servers/hipdb	75 [102]
HIV Drug Resistance DB	No	-	http://hivdb.stanford.ed	935 [56]
HIV Positive Selection Mutation DB	No	-	http://fold.doe-mbi.ucla.edu/HIV/	35[103]
HIVsirDB	No[c]	2011	http://crdd.osdd.net/raghava/hivsir/	45 [104]
HIV Systems Biology	No	-	http://hivsystemsbiology.org	19 [105]
HTLV-1 Molecular Epidemiology DB	Yes	2012[d]	http://htlv1db.bahia.fiocruz.br	13 [106]
HVDB	Yes	2021	http://s2as02.genes.nig.ac.jp	63 [107]
ICTV	Yes	2023	https://ictv.global	929 [30,31]
IRD[a]	Yes	-	http://www.fludb.org	327 [108]
ISED	No	-	http://influenza.cdc.go.kr	19 [109]
IVDBd	No	-	http://influenza.big.ac.cn	74 [110]
LANL HCV Database	Yes	2014	http://hcv.lanl.gov	519 [111,112]
LANL HFV Database	Yes	2015	http://hfv.lanl.gov	26 [113]
LANL HIV Database	Yes	2023	http://hiv.lanl.gov	208 [53,54]
NCBI-HHPID	Yes	2017	http://www.ncbi.nlm.nih.go/RefSeq/HIVInteractions	[93,114,115]
NCBI-IVR	Yes	2023	http://www.ncbi.nlm.nih.gov/genomes/FLU/	1101 [116]
NCBI Viral Genome	Yes	2023	http://www.ncbi.nlm.nih.gov/genomes/VIRUSES/viruses.html	511 [38]
NCBI-VVR	Yes	2023	http://www.ncbi.nlm.nih.gov/genomes	44 [117]
OpenFluDB	Yes	2010[d]	http://openflu.vital-it.ch	45 [118]
PaVE	Yes	2023	http://pave.niaid.nih.gov	285 [58]
PBRC	No[b]		http://www.poxvirus.org	69 [119]
PhEVER	Yes	2010	http://pbil.univ-lyon1.fr/databases/phever/help.html	10 [120]
phiSITE	Yes	2010	http://www.phisite.org/	117[121,122]
RNA Virus Database	No	-	http://tree.bio.ed.ac.uk/rnavirusdb	21 [123]
Subviral RNA Database	No[c]	-	http://subviral.med.uottawa.ca	101 [124]
VBRC	No	-	http://www.vbrc.org/	
VGDB	No	-	http://athena.bioc.uvic.ca/genomes/index.html	35 [125]
VIPERdb	Yes	2023	http://viperdb.scripps.edu	457[33,34]
ViPR[a]	Yes	2023	https://legacy.viprbrc.org/brc/home.spg?decora-tor=vipr	624 [126]
ViralORFeome	No[b]		http://www.viralorfeome.com	48[127]
ViralZone	Yes	2023	http://www.expasy.org/viralzone/	422 [32]
VirHostNet	No	-	http://pbildb1.univ-lyon1.fr/virhostnet	164 [128]
Vir-Mir db	Yes	2007	http://alk.ibms.sinica.edu.tw	106[129]
VirOligo	No	-	http://viroligo.okstate.edu/	28 [130]
VIRsiRNAdb	Yes	2011[d]	http://crdd.osdd.net/servers/virsirnadb	50 [131]
VirusMint	No	-	http://mint.bio.uniroma2.it/virusmint/	219 [132]

Table S2. This table displays the status of virus databases and tools reviewed by Mcleod and Upton in 2017 [23]. For each database, information on availability, last update, URL, and citations is listed. Of the 51 databases mentioned in the original paper under the category sequences databases, only 11 have been updated since 2022.

The number of citations was collected in January 2023. [a]: changed name/URL. "–" = Not Available

Website	Acc	Update	URL	Cite
ACLAME	No	2013	http://aclame.ulb.ac.be/	522 [133,134]
ATIVS	No	-	http://influenza.nhri.org.tw/ATIVS/	18 [135]
AVPdb	No	-	http://crdd.osdd.net/servers/avpdb/	181 [91]
AVPpred	No	-	http://crdd.osdd.net/servers/avppred/	221 [136]
bNAber	No	-	http://bnaber.org/	92 [92]
CAPIH	No	-	http://bioinfo-dbb.nhri.org.tw/capih/	9 [137]
COGs	Yes	2021	http://www.ncbi.nlm.nih.gov/COG/	10519[138–141]
Bat Assoc. Viruses[a]	Yes	2023	http://www.mgc.ac.cn/DBatVir/	14 [43]
euHCVdb	No	-	https://euhcvdb.ibcp.fr/euHCVdb/	165 [96]
euresist	Yes	2023	http://www.euresist.org/web/guest	59 [142]
ExPASy	Yes	-	http://www.expasy.org/	48 [143]
FLAVIdb	No	-	http://cvc.dfci.harvard.edu/flavi/	23 [97]
HBVRegDB	No	-	http://lancelot.otago.ac.nz/HBVRegDB/	38 [99]
HCV DB Project	Yes	2005	http://hcv.lanl.gov/	519 [111,112]
Hepatitis B Virus	No	-	https://hbvdb.ibcp.fr/	184 [57]
Hepatitis Virus DB	Yes	2021	http://s2as02.genes.nig.ac.jp/	63 [107]
HFV/Ebola DB	Yes	2015	http://hfv.lanl.gov/	26 [113]
HIPdb	No	-	http://crdd.osdd.net/servers/hipdb/	79 [102]
HIV Drug Resist.	Yes	2023	http://hivdb.stanford.edu/	1168 [144]
HIV Sequence Database	Yes	2023	http://www.hiv.lanl.gov/	208 [53,54]
HIV-1, Human Protein Interactions	Yes	2017	http://www.ncbi.nlm.nih.gov/genome/viruses/retro-viruses/hiv-1/interactions/	304 [93]
HIVsirDB	No	-	http://crdd.osdd.net/raghava/hivsir/	45 [104]
HTLV-1 Molecular EpidemiologyDB	Yes	2012	http://htlv1db.bahia.fiocruz.br/	13 [106]
Influenza Resource	Yes	2023	http://www.ncbi.nlm.nih.gov/genomes/FLU/	1101 [116]
IRD[a]	Yes	2023	http://www.fludb.org	327 [108]
IRESite	Yes	2019	http://iresite.org/	256 [145,146]
IVDB	No	-	http://influenza.big.ac.cn/	74 [110]
KISED	Yes	2020	http://influenza.cdc.go.kr/	19 [109]
NCBI Genomes	Yes	2023	http://www.ncbi.nlm.nih.gov/genome/viruses/	531 [38]
OpenFlu database	Yes	-	http://openflu.vital-it.ch/	45 [118]
PaVE	Yes	2023	http://pave.niaid.nih.gov/	285 [58]
Phage Genomes	Yes	2015	http://www.ebi.ac.uk/genomes/phage.html	-
PhEVER	Yes	2011	http://pbil.univ-lyon1.fr/databases/phever/	10 [120]
phiSITE	Yes	2014	http://www.phisite.org	117 [121,122]
RNA Virus DB	No	-	http://bioafrica.mrc.ac.za/rnavirusdb/	21 [123]
SARS Cov Res.	Yes	2023	http://www.ncbi.nlm.nih.gov/genomes/SARS/	329 [37]
Subviral RNA	No	-	http://subviral.med.uottawa.ca/	101 [124]
Sugar Bind DB	Yes	2018	http://sugarbind.expasy.org/	45 [147]
Repository for HBV Strain Data	No	-	http://www.hpa-bioinformatics.org.uk/HepSEQ-Research/	-
VaZyMolO	No	-	http://www.vazymolo.org/	45 [148]
Virology.ca	No	-	http://www.virology.ca/	-
VIPERdb	Yes	2023	http://viperdb.scripps.edu/	457[33,34]
Vir-Mir Database	Yes	2007	http://alk.ibms.sinica.edu.tw/	106[129]
ViRAD	Yes	2014	http://www.firthlab.path.cam.ac.uk/virad.html	86 [149]
ViralDB - HUG	Yes	-	http://cegg.unige.ch/viraldb/	-
ViRBase	No	2021	http://www.rnasociety.org/virbase/	9 [150]
VirOligo	No	-	http://viroligo.okstate.edu/	28 [130]
VIRsiRNAdb	No	-	http://crdd.osdd.net/servers/virsirnadb/	50 [131]
ViPR[a]	Yes	2023	http://www.viprbrc.org	624 [126]
Virus Variation	Yes	2017	http://ncbi.nlm.nih.gov/genome/viruses/variation/	329 [37]
VirusMint	No	-	http://mint.bio.uniroma2.it/virusmint/	219 [132]

Table S3. Virus database characteristics are listed, including search features, download options, and methods of accessing the data. **Name**: The name or title of the platform or database. **Keyword Search**: Indicates whether the platform supports keyword search functionality. **Phrase Suggestion**: Indicates whether the platform provides phrase suggestion or auto-complete features. **Cross-Linking Data Pass**: Indicates whether the platform allows cross-linking of data or information to the other databases. **Shareable URL**: Indicates the possibility of access to the same results with sharing the URL address. **WEB API**: Indicates whether the platform generates the web pages via API. **Programmatic Access**: Indicates whether the platform allows programmatic access (API) to its data or services. **Export Results Table**: Indicates any export format of the search result table. **Download Options for Sequence**: Indicates the way to download sequence data (WEB, FTP, or API). **Source-Code Access**: Indicates whether the platform offers access to its source code. **One-Click to Download All**: Indicates whether the user is able to download all the platform data via a simple action. **Download Without Login**: Indicates whether the platform allows downloading without requiring user login or authentication. –: Not Applicable. [a]: CDP-File-Downloader is a tool to download ENA data from the COVID-19 Data Portal. [b]: there are predefined datasets per genotype to download as links.

Name	Keyword Search	Phrase Suggestion	Cross-Linking Data Pass	Shareable URL	WEB API	Export Results Table	Download	Source-Code Access(GitHub)	One-Click to Download	Download Without Login
ICTV	✓	✗	✓	✓	✓	✓	WEB	✓	all	✓
ViralZone	✓	✓	✓	✓	✗	✗	–	✗	–	–
VIPERdb	✓	✗	✓	✓	✓	✓	WEB	✗	one	✓
Virus-Host DB	✓	✗	✓	✗	✗	✓	FTP	✗	all	✓
BV-BRC	✓	✗	✓	✓	✓	✓	WEB, FTP, API	✓	by selection	✓
NCBI Virus	✓	✓	✓	✓	✓	✓	WEB, API	✗	all	✓
NCBI Viral Genomes	✓	✗	✓	✓	✓	✓	WEB, FTP, API	✗	one	✓
RVDB	✗	✗	✗	✗	✗	✓	WEB	✓	all	✓
VOGDB	✓	✗	✓	✓	✗	✗	WEB, FTP	✗	all	✓
Virxicon	✓	✓	✓	✓	✓	✓	WEB, API	✗	by selection	✓
ZOVER	✓	✗	✓	✓	✓	✓	WEB	✗	by selection	✓
IMG/VR	✓	✓	✗	✗	✓	✓	WEB, API	✓	all	✗
MVIP	✓	✗	✓	✓	✗	✗	WEB	✗	one	✓
Viral Host Range DB	✓	✓	✓	✓	✗	✓	–	✗	–	✗
EpiCov (GISAID)	✓	✗	✗	✗	✗	✓	WEB	✗	by selection	✗
The COVID-19 Data Portal	✗	✓	✓	✓	✓	✓	WEB, FTP, API, CDP[a]	✗	all	✓
COVDB	✗	✓	✓	✓	✓	✓	–	✓	–	✓
LANL HIV Database	✓	✗	✓	✗	✗	✗	WEB	✗	by selection	✓
EuResist	–	–	–	–	–	–	–	–	–	✗
HIV Drug Resistance DB	✗	✓	✓	✓	✓	✓	WEB	✓	one[b]	✓
HBVdb	✗	✓	✓	✓	✗	✗	WEB	✗	one	✓
PaVE	✓	✗	✓	✓	✓	✗	WEB, API	✗	by selection	✓
NCBI VVR	✓	✗	✗	✓	✓	✓	WEB, API	✗	by selection	✓
PSD	✗	✓	✓	✗	✗	✓	WEB	✗	all	✓

Table S4. Here, all current coronavirus databases are listed.

**Coronavirus databases**
RCoV19
ICTRP
CoVDB
SARS-CoV-2 related structures
RNAStructuromeDB
SARS-CoV-2 MAT
CoronaCentral
ESC
DBCOVP
Ensembl COVID-19
Coronavirus GenBrowser
COKE
KGCoV
hCoronavirusesDB
SARS-CoV-2 Database
COVID-19 Data Portal Spain
COVID-19 SeroHub (SeroHub
National Center for Advancing Translational Sciences COVID-19 OpenData Portal
The WHO Global Clinical Platform for COVID-19
Consortium for Clinical Characterization of COVID-19 by EHR(4CE)
ASH RC COVID-19 Registry for Hematology
Pregnancy CoRonavIrus Outcomes RegIsTrY (PRIORITY)
COVID-19 Registry
The COVID-19 and Cancer Consortium (CCC19)
COVID-19 CVD Registry
COVID-19 Dermatology registry
Global Registry of COVID-19 in Pediatric Cancer
MS Global Data-Sharing Initiative
ASCO Survey on COVID-19 in Oncology (ASCO) Registry
Discovery VIRUS COVID-19 Registry
Dutch National COVID-19 metadata portal(COVID-NL metadata)
Dutch National COVID-19 clinical data portal (COVID-NL clinical data)
COVID-19 Host Genetics Initiative (COVID-19 hg)
Canadian VirusSeq Data Portal(CVDP)
Database of publications on coronavirus disease (COVID-19)
Surveillance Epidemiology of Coronavirus (COVID19) Under Research Exclusion
NCBI SARS-CoV
COViMS
Surveillance Epidemiology of Coronavirus (COVID19) Under Research Exclusion
Biobanque québécoise de la COVID-19
National COVID Cohort Collaborative
John Hopkins Coronavirus Resource
LitCovid
The COVID-19 Data Portal
CORDITE
VirHostNet 3.0

Table S5. The table displays the full evaluation of the FAIR criteria and includes the data source used for the databases listed in the current review. Please refer to the S1 for the full descriptions of each FAIR subcriteria. Note that some of the databases were not included in the FAIR evaluation due to the lack of a comparable table. The URLs behind the database names are clickable and lead to the data sources from each databases. 0=no, 1=yes.

Name	F1	F2	F3	F4	A1	A1.1	A1.2	A2	I1	I2	I3	R1	R1.1	R1.2	R1.3
ICTV	0	1	1	0	0	0	1	1	0	1	1	1	0	1	1
ViralZone	0	1	0	1	0	1	1	1	0	1	1	1	1	1	1
VIPERdb	1	1	1	1	1	1	1	1	0	0	1	1	0	1	1
Virus-Host DB	1	1	1	1	1	1	1	1	0	0	1	1	1	1	1
BV-BRC	1	1	1	1	1	1	1	1	0	0	1	1	0	1	0
NCBI Virus	1	1	1	0	1	1	1	1	0	1	1	1	0	1	1
NCBI Viral Genomes	–	–	–	–	–	–	–	–	–	–	–	–	–	–	–
RVDB	0	1	0	0	0	0	1	1	0	0	0	0	0	1	0
VOGDB	1	1	1	0	1	1	1	1	0	0	1	1	0	1	0
Virxicon	0	1	0	0	0	1	1	0	0	0	1	1	0	0	0
ZOVER	0	1	0	0	0	0	0	1	0	0	1	1	0	0	0
Viral Host Range DB	0	1	1	0	1	1	1	1	0	0	1	1	0	1	0
IMG/VR v4	1	1	1	1	1	1	1	1	0	1	1	1	0	1	1
MVIP	1	1	1	0	1	1	1	1	0	0	1	1	0	1	0
EpiCov (GISAID)	1	1	1	1	1	1	1	1	0	0	1	1	1	1	0
Covid-19 Data Portal	1	1	1	1	1	1	1	1	1	0	1	1	1	1	1
COVDB	0	1	0	0	0	0	0	1	0	0	1	1	0	0	1
LANL HIV Database	0	1	1	0	0	0	0	1	0	1	1	1	0	1	0
EuResist	–	–	–	–	–	–	–	–	–	–	–	–	–	–	–
HIV Drug Resistance DB	0	1	0	0	0	0	0	1	0	1	1	1	0	1	1
HBVDB	0	1	1	0	0	1	1	0	0	1	0	1	0	1	0
PaVE	1	1	1	0	1	1	1	1	0	0	1	1	1	1	0
NCBI VVR	1	1	1	0	1	1	1	1	0	1	1	1	0	1	1
PSD	0	1	0	0	1	1	1	1	1	0	1	0	0	0	0

Table S6. Here, various types of errors that can occur in the naming of viruses are shown, along with an example.

Error type	official name	additional names
Change of name	Kuttervirus CBA120	Escherichia virus CBA120
Name extension	Alphasphaerolipovirus HCIV1	Haloarcula virus HCIV1
Abbreviations	Chimeric virus 14	CHIV14
Spelling mistake	Privet ringspot virus	Privet ringsport virus
Capitalization	Escherichia phage Andreotti	Escherichia phage andreotti
Character error	Human T-cell leukemia virus type I	Human T cell leukemia virus type 1

Table S7. This table provides an overview of the metadata availability for 9,763,946 virus genomes in the `BV-BRC` database. Each column corresponds to a specific metadata category, and the reported quantity represents the total count of records containing any value, including unclassified and undefined values. The selection of columns in this table was made from the available genome metadata in `BV-BRC`, considering their likelihood of providing comprehensive information suggested by Wagner *et al.* in 2021 [151] and Field *et al.* in 2008 [152]. The table suggests that in 71.43 % of the minimum metadata categories (10 out of 14 columns), `BV-BRC` provides data for over 50 % of the records.

Column Name	Quantity	Percentage
Collection Attributes		
Collection Year	8,991,879	92.09 %
Isolation Country	9,162,987	93.85 %
Isolation Source	9,763,625	~100.00 %
Host Common Name	9,151,228	93.72 %
Database Crosslinks		
GenBank Accession	9,607,314	98.40 %
Taxon Lineage ID	9,763,946	100.00 %
Publication	830,653	8.51 %
Species Variations		
Lineage	6,651,009	68.12 %
Strain	6,765,991	69.30 %
Subtype	961,142	9.84 %
Segment	1,102,852	11.30 %
Sequence Information		
Genome Length	9,735,932	99.71 %
Genome Quality	94,154	0.96 %
Genome Status	9,748,928	99.85 %

Figure S1. FAIR Evaluation Criteria from Reference with additional guidelines

Here the FAIR criteria are listed below. This information is exactly reproduced from the FAIR Principles website which can be found at https://www.go-fair.org/fair-principles/. Our interpretation of the FAIR criteria was enhanced by referring to the GoFAIR definitions, which can be found at https://www.gofair.foundation. **Findable.** The first step in (re)using data is to find them. Metadata and data should be easy to find for both humans and computers. Machine-readable metadata are essential for automatic discovery of datasets and services, so this is an essential component of the FAIRification process.

F1 (Meta)data are assigned a globally unique and persistent identifier
F2 Data are described with rich metadata (defined by R1 below)
F3 Metadata clearly and explicitly include the identifier of the data they describe
F4 (Meta)data are registered or indexed in a searchable resource

Accessible. Once the user finds the required data, she/he/they need to know how they can be accessed, possibly including authentication and authorisation.

A1 (Meta)data are retrievable by their identifier using a standardised communications protocol
A1.1 The protocol is open, free, and universally implementable
A1.2 The protocol allows for an authentication and authorisation procedure, where necessary
A2 Metadata are accessible, even when the data are no longer available

Interoperable. The data usually need to be integrated with other data. In addition, the data need to interoperate with applications or workflows for analysis, storage, and processing.

I1 (Meta)data use a formal, accessible, shared, and broadly applicable language for knowledge representation.
I2 (Meta)data use vocabularies that follow FAIR principles
I3 (Meta)data include qualified references to other (meta)data

Reusable. The ultimate goal of FAIR is to optimise the reuse of data. To achieve this, metadata and data should be well-described so that they can be replicated and/or combined in different settings.

R1 (Meta)data are richly described with a plurality of accurate and relevant attributes
R1.1 (Meta)data are released with a clear and accessible data usage license
R1.2 (Meta)data are associated with detailed provenance
R1.3 (Meta)data meet domain-relevant community standards

The principles refer to three types of entities: data (or any digital object), metadata (information about that digital object), and infrastructure. For instance, principle F4 defines that both metadata and data are registered or indexed in a searchable resource (the infrastructure component).

Additional guidelines

For the current FAIR evaluation, the following guidelines were used to determine the FAIR scores of 1:compliance, and 0:no compliance: **Evaluation Notes: F1.** If the ID was INSDC and the database was INSDC this counts as yes; if it was something like "1,2,3" then no; **F2.** If metadata fields > 3 then yes; **F3.** If there was an id (not necessarily a global and persistent id) there then yes; **F4.** The database was considered the searchable resource; if there was a search bar and the entry could be found by id then yes; **A1.** If metadata available by the id (e.g. summary page and/or downloadable cvs etc) then yes; **A1.1.** (e.g. clicking a link); **A1.2.** If referring to public data resources, yes; **A2.** If publication, name, BioProjectID or other accession id was included in metadata then yes; **I1.** If metadata was in a formal language then yes; **I2.** If metadata used explicitly stated vocabularies then yes; or if the db was an official organization (NCBI/ENA/ICTV) and the metadata came from within then yes; **I3.** f links in metadata then yes; **R1.** If metadata fields > 3 then yes; **R2.** If license linked then yes; **R3.** If in publication or on website then yes; **R4.** Lack of community standards;

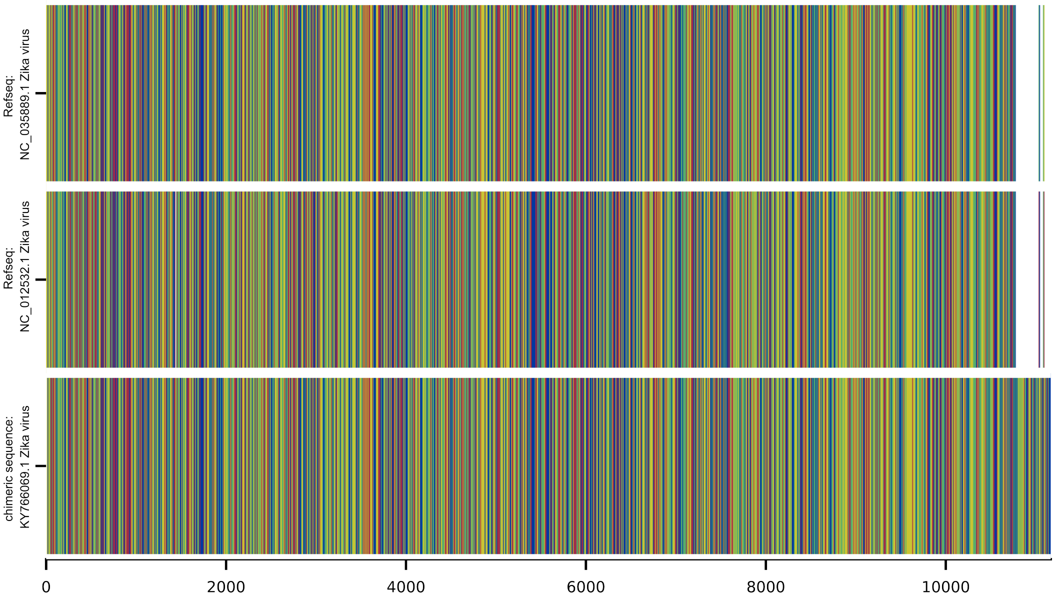

Figure S2. A schematic representation of the full genome alignment between the sequence of interest below and the two reference sequences of Zika (above) is shown here. The alignment was constructed using `Mafft` (v7.310) and visualized with `CIAlign`(1.0.18) [153,154]. Note that here the 3′ end of the sequence of interest does not match with the reference sequences.
